# Camouflaged Fluorescent Silica Nanoparticles Target Aggregates and Condensates of the Amyloidogenic Protein Tau

**DOI:** 10.1021/acs.bioconjchem.2c00168

**Published:** 2022-06-10

**Authors:** Carlo Giorgio Barracchia, Francesca Parolini, Angela Volpe, Daniele Gori, Francesca Munari, Stefano Capaldi, Mariapina D’Onofrio, Michael Assfalg

**Affiliations:** † Department of Biotechnology, University of Verona, 37134 Verona, Italy; ‡ ACZON srl, Monte San Pietro, BO 40050, Italy

## Abstract

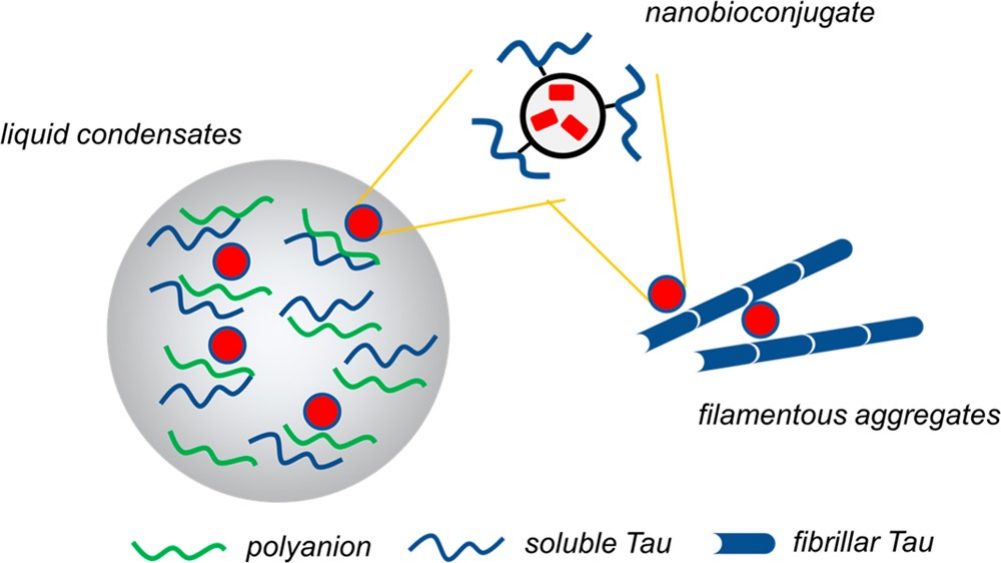

Intrinsically disordered proteins (IDPs) are increasingly found to be associated with irreversible neurodegenerative disorders. The protein tau is a prototypical IDP whose abnormal aggregation into insoluble filaments is a major hallmark of Alzheimer’s disease. The view has emerged that aggregation may proceed via alternative pathways involving oligomeric intermediates or phase-separated liquid droplets. Nanoparticles (NPs) offer significant potential for probing the mechanisms of protein fibrillation and may be capable of redirecting conformational transitions. Here, we camouflaged dye-doped silica NPs through functionalization with tau molecules to impart them the ability to associate with protein assemblies such as aggregates or condensates. The prepared NP–tau conjugates showed little influence on the aggregation kinetics and morphology of filamentous aggregates of tau but were found to associate with the filaments. Moreover, NP–tau conjugates were recruited and concentrated into polyanion-induced condensates of tau, driven by multivalent electrostatic interactions, thereby illuminating liquid droplets and their time-dependent transformation, as observed by fluorescence microscopy. NP–tau conjugates were capable of entering human neuroglioma cells and were not cytotoxic. Hence, we propose that NP–tau conjugates could serve as nanotracers for in vitro and in-cell studies to target and visualize tau assemblies and condensates, contributing to an explanation for the molecular mechanisms of abnormal protein aggregation.

The aberrant aggregation of peptides and proteins into amyloid fibrils and their accumulation into insoluble deposits in the brain are the hallmarks of increasingly widespread neurodegenerative conditions, such as Parkinson’s (PD) and Alzheimer’s diseases (AD).^1^ A number of proteins implicated in these disorders, including α-synuclein (αSyn) and the microtubule-associated protein tau, are largely unstructured in solution and hence are described as natively unfolded or intrinsically disordered.^2^ The molecular mechanism by which these soluble polypeptides convert into small aggregates and into higher-order supramolecular assemblies has been intensely investigated, although not fully clarified.^3^ The view has emerged that the conformational transition from normal to amyloid states can proceed alternatively via a deposition pathway involving oligomeric intermediates or via a condensation pathway involving phase-separated liquid droplets.^4^

Interfering with either aggregation mechanism has been envisioned as a promising disease-modifying approach for the treatment of AD, PD, and other proteinopathies.^5^ However, the disordered nature of the implicated proteins precludes the use of structure-based drug design approaches for the discovery of molecules able to modulate protein aggregation. Small-molecule inhibitors of protein aggregation have shown limited success, possibly due to the lack of specificity and to the complex and dynamic nature of the protein aggregation intermediates.^6^ Nanoparticles (NPs), with their large surface area available for protein adsorption and their small size which facilitates access to tissues and cells, offer significant potential for probing the mechanisms of protein fibrillation and, in the longer term, for treatment of amyloidogenic diseases.^7^

Previous studies have shown the ability of NPs to inhibit and even reverse the formation of amyloid fibrils.^8−10^ Such an effect was attributed to monomer depletion and/or trapping of sub- and near-critical nuclei.^8^ On the other hand, NPs interacting with amyloidogenic proteins may catalyze protein aggregation by a mechanism of surface-assisted nucleation.^11,12^ Simple formulations of NPs of varied materials have been extensively explored in a bid to unravel the surface-mediated conformational transitions associated with protein aggregation.^13−15^ The next challenge is to determine how more specific NP-surface decorations interact with amyloidogenic proteins. In this regard, an earlier study reported the bioactivity of NPs functionalized with the amyloid protein itself.^16^ Quantum dots multifunctionalized with αSyn were found to significantly accelerate αSyn fibrillation and were proposed as nanoactuators for in-cell studies of protein aggregation.

Here, we prepared a nanoconjugate based on silica NPs functionalized with the microtubule-binding domain of tau. Tau is predominantly an intracellular soluble protein that associates with and stabilizes microtubules in neuronal axons.^17^ Abnormal accumulation of tau is associated with AD, frontotemporal dementia (FTD), and other neurodegenerative disorders collectively referred to as tauopathies.^18^ The lysine-rich, four-repeat region (tau^4RD^) of the microtubule-binding domain (Supporting Information Figure S1) contains two hexapeptide motifs that are considered critical aggregation nuclei and are involved in the formation of pathological paired helical filaments.^19^ The isolated tau^4RD^ is a commonly used model system for in vitro aggregation studies. Thus, after the preparation and characterization of the nanoconjugate, we aimed to verify its impact on tau^4RD^ aggregation, its cytotoxicity, and its cellular uptake.

We focused on dye-doped core–shell silica NPs (hereafter simply referred to as NPs) due to their inherent versatility and biocompatibility which make them highly attractive for biomedical applications.^20^ NPs were prepared via micelle-assisted synthesis and consisted of a silica core and a PEG shell.^21^ A fluorescent rhodamine derivative was incorporated into the silica core during preparation. The NPs exhibited maximum fluorescence intensity at 586 nm (Figure 1A) and maximum absorbance at 566 nm (Figure 1B). Colloidal solutions of NPs displayed a narrow size distribution with a mean hydrodynamic diameter of 22.44 ± 0.04 nm, as determined by dynamic light scattering (Figure 1C). NPs were activated by maleimide groups (ca. 10/particle) for conjugation to the sulfhydryl-containing tau molecule. To obtain best homogeneity of the conjugate product and to avoid undesired disulfide bond formation, one of the two native cysteine residues in tau^4RD^ was replaced by alanine [tau^4RD^(C322A)], while Cys291 remained available for conjugation. The NPs and protein were allowed to react at room temperature, and the progress of the reaction was followed by SDS-PAGE (Figure 1D). The product did not enter the running gel; however, it was detectable in the stacking gel, where smeared bands appeared almost immediately after mixing components (Figure 1D, lanes 1–5). The presence of NPs was confirmed by the luminescence produced under UV-lamp irradiation (Figure 1D, right panel). Dimeric tau^4RD^(C322A) species were formed in the absence of reducing agents (Figure 1D, lane 2, MW ∼ 30 kDa) and dissociated after addition of DTT (Figure 1D, lane 3). The desired product (NP–tau) was successfully purified from unreacted tau^4RD^ by dialysis (Figure 1D, lane 4). The absorption spectrum of the purified nanoconjugate (NP–tau) showed slightly increased intensity around 280 nm, compared to NPs (Figure 1B), ascribed to the single tyrosine residue of conjugated tau^4RD^(C322A). Based on absorbance measurements, we estimated an average stoichiometry of ca. 8 protein molecules per particle.

**Figure 1:**
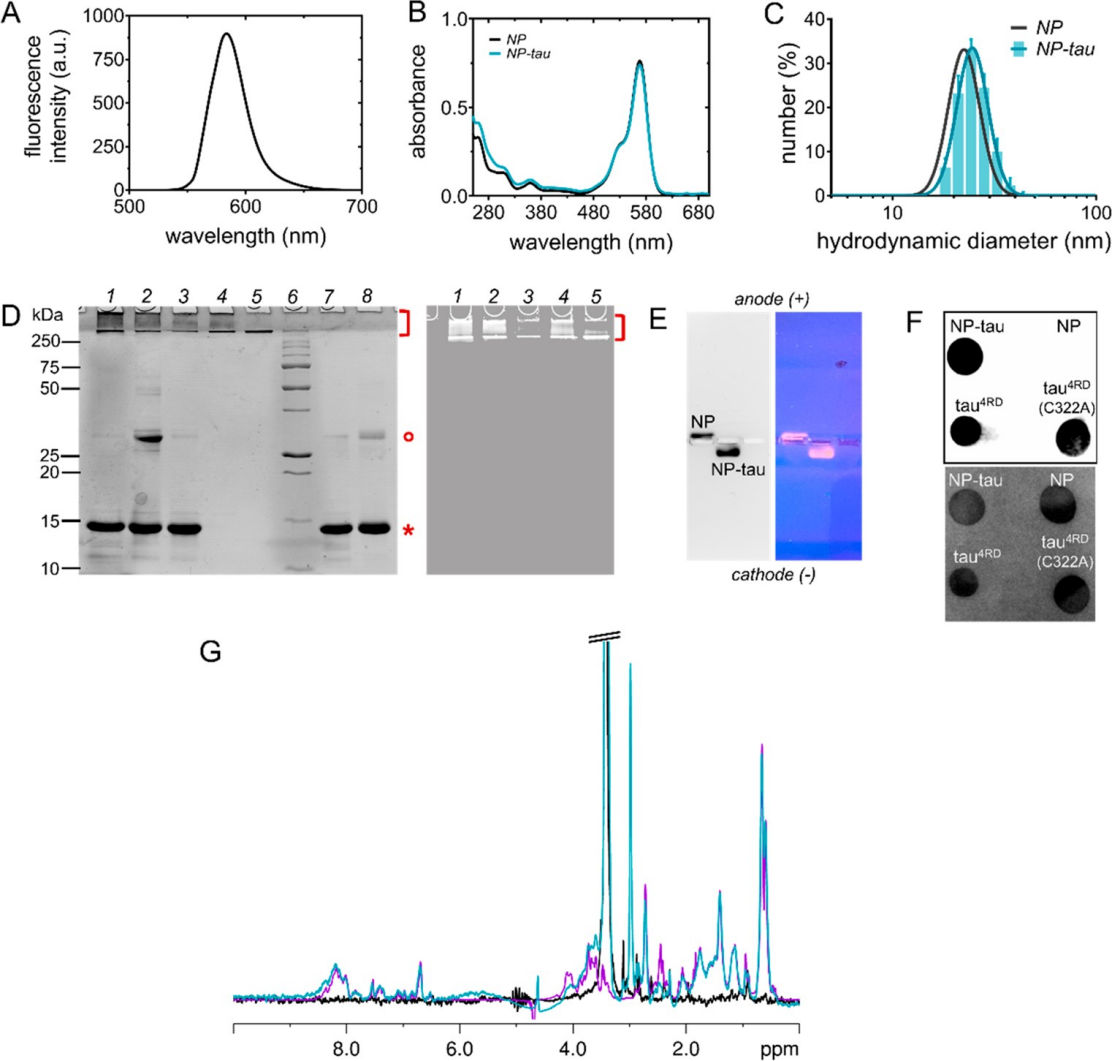
Characterization of the protein–NP conjugate. (A) Fluorescence emission spectrum (λ_ex_ = 565 nm) of dye-doped NPs. (B) UV–visible absorption spectrum of the unconjugated NP (black) and NP–tau (marine blue). (C) Hydrodynamic diameter distribution of nanoparticles as determined from dynamic light scattering; continuous lines are the best-fit Log–Gaussian curves for the unconjugated NP (black) and NP–tau (marine blue); bars are displayed for NP–tau only, for better visualization. Error bars are s.d. of replicate measurements (*n* = 5). (D) SDS-PAGE analysis of the conjugation reaction of NPs with tau^4RD^(C322A); left panel displays the gel after Coomassie staining, and the right panel displays the same gel visualized under UV light. Lanes correspond to (1) reaction mixture at *t* = 0; (2) reaction mixture at *t* = 4 h; (3) reaction mixture at *t* = 4 h followed by addition of DTT; (4) purified NP–tau nanoconjugate; (5) NPs; (6) molecular weight marker; (7) tau^4RD^(C322A); (8) tau^4RD^; red brackets: NP–tau; red circle: tau^4RD^ dimers; red asterisk: tau^4RD^ monomer. (E) Agarose gel electrophoresis of the NP–tau nanoconjugate visualized by Coomassie stain (left) and under UV light (right); control sample contains unconjugated NPs. (F) Dot blot for detection of tau (anti-tau359–373) in the NP–tau nanoconjugate; control samples contained NP, tau^4RD^, or tau^4RD^(C322A); samples were also stained with Ponceau dye (bottom panel). Note that unconjugated NPs adsorbed the Ponceau. (G) Overlaid ^1^H NMR spectra of: the tau^4RD^(C322A) (magenta), NP (black), and NP–tau (marine blue). Spectra were acquired at 10 °C, and cut signals are from EDTA.

After protein conjugation, the hydrodynamic diameter of NPs expanded to 24.67 ± 0.05 nm. Furthermore, the functionalization modified the surface charge of the NPs from slightly negative (electrokinetic surface potential, ζ = −5.68 ± 0.93 mV) to positive values (ζ = 5.0 ± 0.7 mV), as expected given the highly basic character of tau^4RD^ (pI = 9.7, ζ = 21.7 ± 1.0 mV). Consistent results were obtained by agarose gel electrophoresis, where unconjugated NPs migrated slightly toward the anode, and NP–tau migrated in the opposite direction toward the negative electrode (Figure 1E). Immunoblot analysis performed with the antitau antibody confirmed the presence of tau^4RD^(C322A) conjugated to NPs (Figure 1F). The NMR spectrum of purified NP–tau suggested that conjugated tau remained disordered and flexible, and the few spectral differences with respect to unbound tau were likely due to the acquired local rigidity near the anchoring site (Figure 1G).

Following the characterization of NP–tau, we set to explore its activity on tau filament formation. The aggregation kinetics of tau^4RD^, stimulated by addition of the cofactor heparin, was followed by monitoring the time-dependent fluorescence signal of Thioflavin-T (ThT), a benzothiazole dye responsive to the cross-β structures characteristic of amyloid fibrils.^22^ The kinetic profile was sigmoidal shaped and consistent with a macroscopic nucleation–growth mechanism, in both the absence and presence of NP–tau (Figure 2A). The presence of NP–tau had a modest influence on the kinetics of aggregation, resulting in a slightly accelerated transition (midpoint transition time constant, *t*_0.5_ = 1.0 h vs 3.2 h in the absence of NPs) and slightly faster fibril elongation rate (elongation time constant, τ = 1.8 h vs 2.1 h). Conjugated tau did not form ThT-positive structures in the absence of unconjugated protein (Figure 2A). We further investigated the conformational changes of tau^4RD^ by collecting circular dichroism (CD) spectra at different time points (Figure 2B). At the start of the incubation period, the spectrum displayed a typical profile of a prevalently disordered polypeptide. After 24 h of incubation in aggregating conditions, the spectral profile converted into one mostly reflecting β-structure motif (Figure 2B, left). Only minor changes were observed after a further 24 h of incubation, consistent with the ThT fluorescence data which indicated that a steady state was reached before 24 h, when most of the tau molecules had transformed into fibrillar species. The CD spectra collected in the presence of NP–tau displayed no significant difference from the corresponding spectra obtained in the absence of a nanoconjugate (Figure 2B, right). Using transmission electron microscopy (TEM), we examined the aggregation products and observed the formation of long filaments with similar morphology in the absence and presence of NP–tau (Figure 2C,D). Hence, the NP–tau nanoconjugates did not significantly perturb tau filament formation via the deposition pathway, suggesting that any interaction with aggregation intermediates was weak and reversible, thus scarcely affecting the aggregation process. By contrast, observations from a sedimentation assay and TEM micrographs of thoroughly washed deposits (Figure 3) indicated that NP–tau stably associated with insoluble tau filaments and that the interaction was mediated by the conjugated protein. The association of NP–tau with filaments was observed when NP–tau was added at the beginning and at the end of the aggregation period, with a more marked effect in the former case. It appears that tau filaments were able to recruit NP–tau on their surface; however, it cannot be excluded that conjugated tau was partly incorporated into the filament cores during maturation. Indeed, while the site of conjugation, in the middle of the R2 repeat, is within the core of heparin-induced filaments,^23^ the latter are highly polymorphic and could tolerate the presence of bound NPs. Furthermore, the R2 repeat is absent from the core of tau fibrils derived from AD brains^24^ and is not essential for tau aggregation in vitro.^25,26^

**Figure 2:**
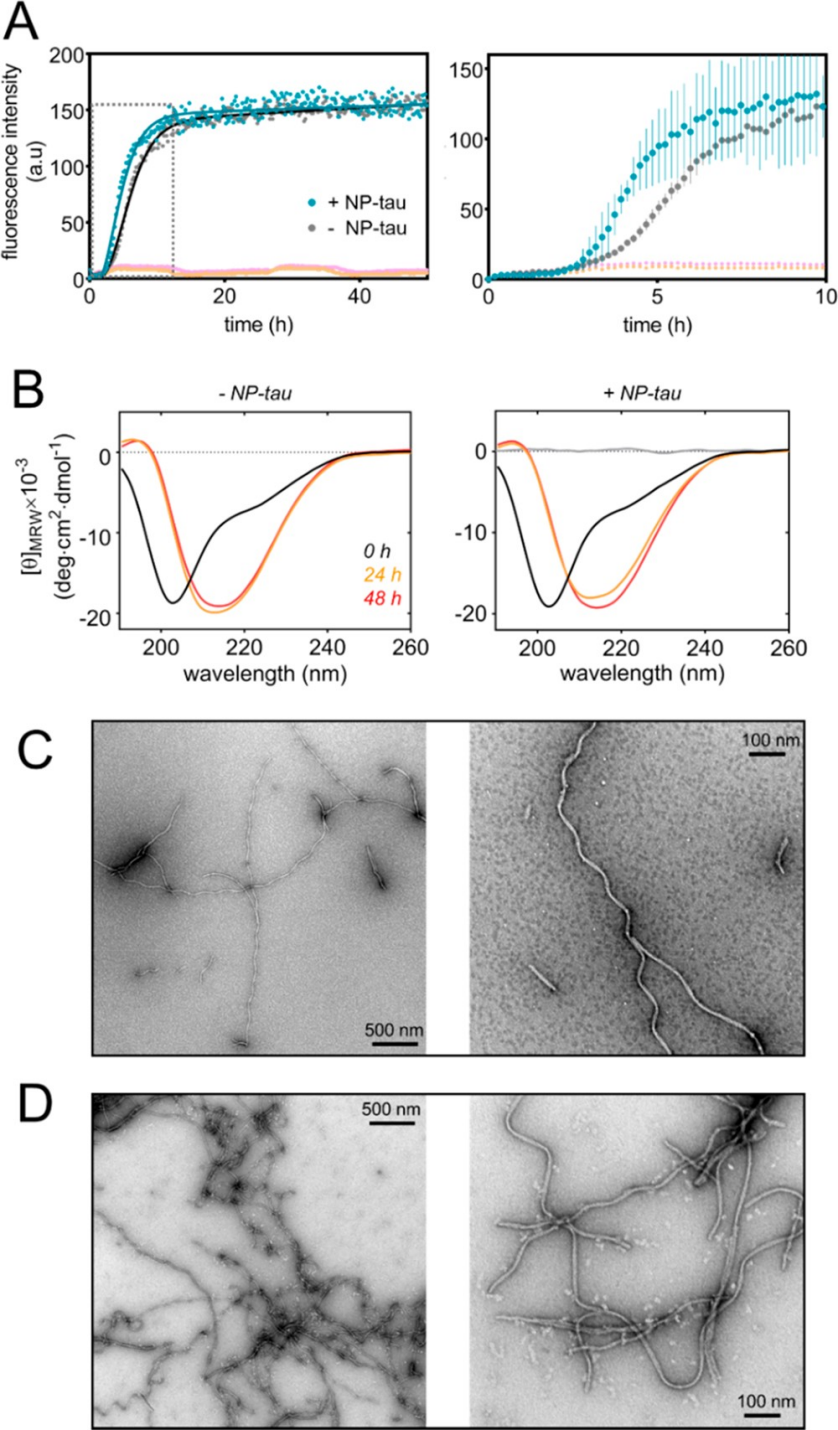
Protein aggregation assays. (A) Aggregation kinetics monitored by ThT fluorescence. Measurements were performed at 30 °C on 10 μM tau^4RD^ in the absence (gray) or presence (marine blue) of NP-tau, and solid lines correspond to the best-fit curves determined using an empirical sigmoid function. Control samples contained NP–tau (orange) and tau^4RD^+NP without an aggregation inducer (pink). Right panel: enlarged view of the lag and growth phases. Data represent the mean ± SD, *n* = 3 replicate measurements. (B) Far-UV CD spectra recorded at 25 °C on 6 μM tau^4RD^ in the absence (left) or presence of NP–tau (right) at various time points of the aggregation process. The signal of NP–tau alone (gray, right panel) is undetectable, likely due to scattering effects near the NP surface and/or heterogeneity of bound-state conformations. (C,D) Representative TEM images of tau^4RD^ filaments formed after 48 h incubation in aggregating conditions in the absence (C) or presence (D) of NP–tau. The molar ratio of unconjugated:conjugated tau was 1:0.5 in all experiments.

**Figure 3:**
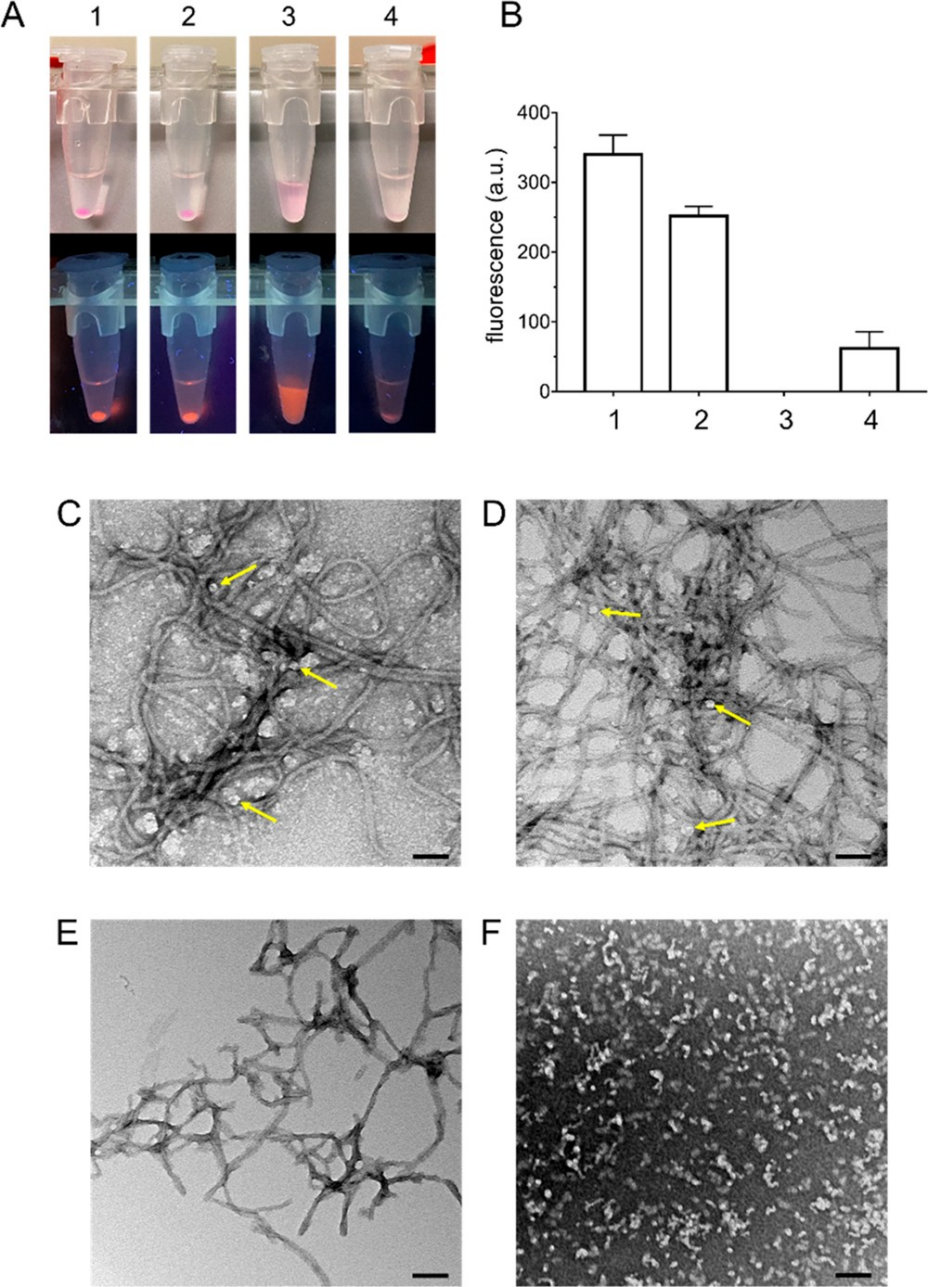
Association of NP–tau with tau aggregates. (A) Sedimentation assay performed on samples of tau^4RD^ and NPs; samples were incubated under aggregating conditions for 24 h at 37 °C without agitation. The solutions were then centrifuged and washed repeatedly and finally examined under daylight (top) or UV light (bottom). Samples contained: (1) tau^4RD^ and NP–tau added at *t* = 0; (2) tau^4RD^, NP–tau added at *t* = 24 h and incubated for 1 h; (3) no protein, NP–tau, shown is the solution after the first centrifugation step; (4) tau^4RDΔC^, unconjugated NP, samples were prepared using 150 μM protein and 9 μM nanoparticles. (B) Fluorescence measurements on resuspended protein/NP deposits; the pellets resulting from the sedimentation experiment were dispersed in buffer and examined by fluorescence spectroscopy. Data are the mean ± sd from experiments performed in triplicate; labels refer to conditions described for panel A. (C–F) TEM images of tau^4RD^ and NP–tau; micrographs are from (C) filaments of tau^4RD^ formed in the presence of NP-tau; (D) filaments of tau^4RD^ formed in the absence of NP–tau, and the latter were added at *t* = 24 h; yellow arrows point to representative protein-associated NP–tau; (E) filaments of tau^4RDΔC^ formed in the presence of NP; (F) NP–tau; scale bars are 100 nm.

The formation of biomolecular condensates has emerged as a crucial mechanism of the regulation of various biological processes.^27^ Cellular condensates are micron-sized, dynamic assemblies characterized by multivalent intermolecular contacts.^28−32^ They are thought to form through liquid–liquid phase separation (LLPS)^33−37^ and exhibit liquid-like properties (hence the term liquid droplets).^30,38−40^ Condensates are metastable and may transition from liquid to solid-like states as a consequence of aging or pathological insults.^31^ For an increasing number of amyloidogenic proteins, including tau, aberrant LLPS has been associated with pathological aggregation and neurodegeneration.^41−45^ Here, we explored if NP–tau could interact with tau condensates. The LLPS of tau^4RD^ is facilitated by polyanionic cofactors which establish multivalent electrostatic interactions with the polycationic protein. Notably, lysine residues, which are considered critical regulators of biomolecular condensation,^46^ are very abundant in tau^4RD^ (ca. 15% of all amino acids). By mixing tau^4RD^ with polyuridylic acid (polyU) at a molar ratio corresponding to approximately overall net charge neutrality, the solution underwent LLPS (visually detectable by increased sample turbidity). We introduced Alexa-labeled tau^4RD^ as a fluorescent reporter molecule. Fluorescence microscopy images showed the formation of spherical droplets of concentrated protein (Figure 4A) with a mean diameter of 1.7 μm (Figure 4D). Liquid droplets of similar size were also formed in the presence of NPs (Figure 4B,E) and of NP–tau (Figure 4C,F). Different from unconjugated NPs, however, NP–tau was recruited and concentrated into the droplets, as evident from the emitted fluorescence observed in the red channel of the microscope that is associated with the rhodamine dye-doped silica NPs. Thus, conjugated tau was able to establish multivalent interactions within the dense phase, in analogy with the unbound protein. In fact, despite the modification in repeat R2, there is a high number and near-uniform distribution of basic residues throughout the polypeptide available for multivalent binding.^47^

**Figure 4:**
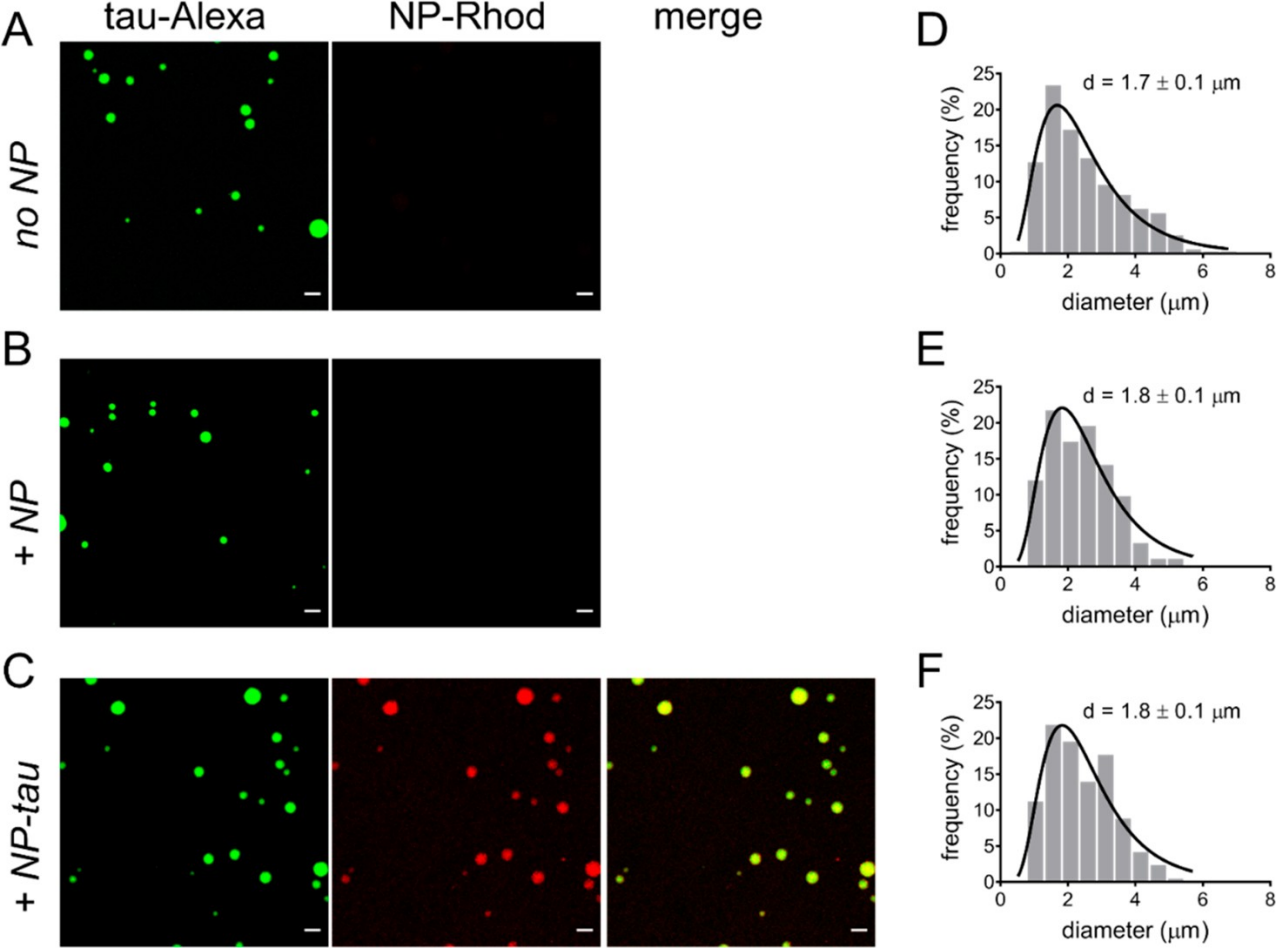
Targeting liquid condensates. (A–C) Representative fluorescence microscopy images of condensates of tau^4RDΔC^/poly(U) prepared in the presence of (A) no particles, (B) unconjugated NPs, and (C) NP–tau. Images were acquired 15 min after preparation, and 25 μM protein was mixed with 62.5 μg/mL of poly(U) RNA in 25 mM Hepes, at pH 7.4. NP–tau was 2.5 μM in conjugated protein. Scale bars are 5 μm. Left: green channel, middle: red channel, right: merge. (D–F) Size distribution of droplet diameters, as determined from acquired micrographs; *d* = peak diameter ± s.e. (*n* = 100–200). Determined from log-normal best-fit curves (black lines).

In a previous study, we found that polyU promoted the formation of relatively stable coacervates without inducing concomitant protein aggregation during an observation period of two to three days.^47^ By contrast, coacervates formed in the presence of heparin, a known aggregation inducer, rapidly evolved into nonspherical assemblies, making them a model system for condensation-linked pathological aggregation.^47^ Thus, we investigated the interaction of NP–tau with heparin-induced condensates. Microscopy images showed that NP–tau was recruited into freshly prepared heparin-containing liquid droplets and illuminated their time-dependent transformation into irregularly shaped assemblies (Figure 5). We conclude that the disordered character, the repetition of sequence motifs, and the distribution of positively charged residues in the conjugated tau domain confer the ability to establish multiple transient intermolecular interactions with the polyanionic components and to promote the recruitment and concentration of the NPs in dense phases.

**Figure 5:**
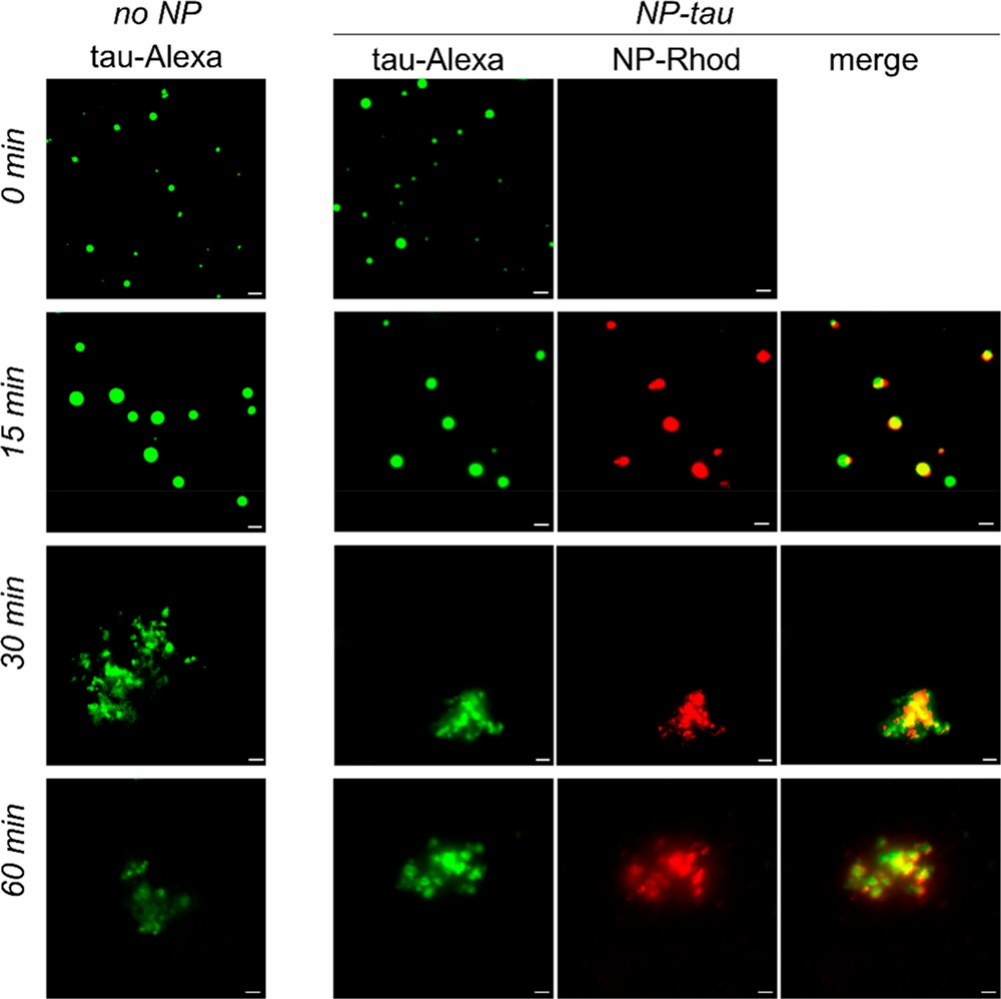
Visualizing droplet transformation. Representative fluorescence microscopy images of condensates of tau^4RDΔC^/heparin prepared in the absence (left) or presence (right) of NP–tau. Images were acquired immediately after addition of protein and after 15, 30, and 60 min of incubation. Scale bars are 5 μm. Samples contained 25 μM protein and 6.25 μM heparin in 20 mM sodium buffer and 30 mM NaCl, at pH 6; NP–tau was 2.5 μM (conjugated protein). Recruitment of NP–tau into heparin-induced condensates showed some delay. Control micrographs for condensates in the presence of unconjugated NPs are shown in Supporting Information Figure S2.

Because cellular condensates are often complex coacervates containing proteins and RNA,^8^ as in our simplified system, the above observations suggest the possibility to exploit NP–tau nanoconjugates to trace the formation, evolution, or dissolution of cellular condensates in models of pathology. The latter possibility stimulated us to verify two fundamental requirements for this potential application: (i) the biocompatibility of NP–tau and (ii) their ability to get internalized into live neuronal cell models. First, we used flow cytometry to quantify cellular uptake of NPs by H4-swe neuroglioma cells. The analysis of the population distribution and mean fluorescence intensity of H4-swe cells treated with different concentrations of NPs (Figure 6A) revealed a clear dose-dependent uptake by this cell line. Next, the cellular internalization of NPs and NP–tau in H4-swe cells was examined by confocal microscopy. Figure 6B shows representative confocal microscopy images of H4-swe cells treated with unconjugated and tau-conjugated NPs. Both were efficiently internalized and were visible as granular aggregates (red dots) in the cytoplasm, particularly in the perinuclear region, while they appeared unable to enter the nucleus. The latter behavior may be a consequence of their size, which prevents their free diffusion across the nuclear membrane through the nuclear pore complexes (NPCs).^48,49^ Previous studies linked nuclear localization of small NPs to increased cytotoxicity since they cannot be easily cleared from cells.^50,51^ Conversely, larger nanoparticles remain in the cytoplasm and can be eliminated by the cells more rapidly. Thus, we tested the biocompatibility of our NPs in H4-swe cells, based on the Trypan Blue exclusion dye assay. As shown in Figure 6C, the vitality of H4-swe cells after 48 h treatment with NPs or NP–tau was not significantly different from that of untreated cells (one-way ANOVA), demonstrating that both samples were not cytotoxic in the tested concentration range.

**Figure 6:**
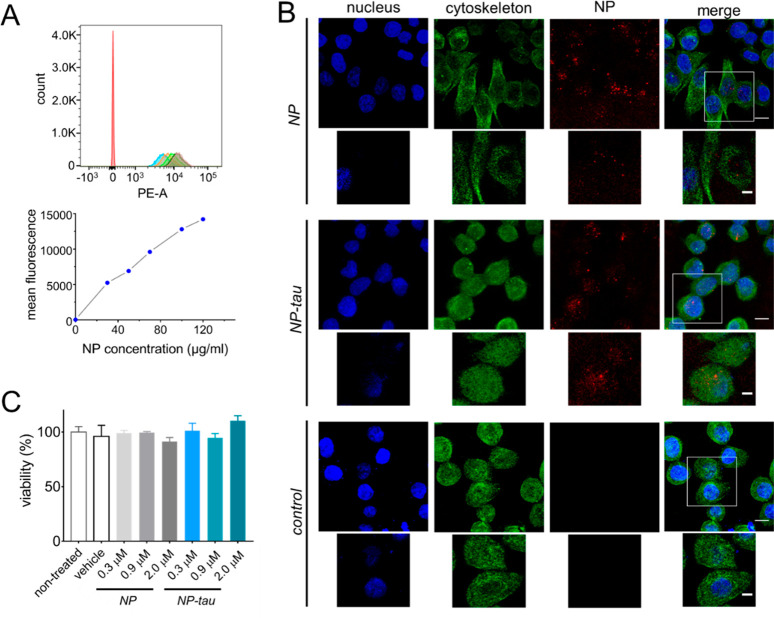
Cellular assays. (A) Top: flow cytometry analyses of H4-swe cells after 48 h treatment with different concentrations of NP (30, 50, 70, 100, and 120 μg/mL). Red: non treated; bottom: mean fluorescence intensity values of cells treated with NP, highlighting the dose-dependent response. (B) Representative confocal microscopy images of H4-swe cells after treatment with 2 μM NP and NP–tau (rhodamine-doped, red) for 48 h. β-Tubulin (cytoskeleton) is stained in green; cell nuclei are stained with Hoechst 33342 (blue). Scale bars are 10 μm. Insets show a single *z*-plane, and scale bars are 5 μm. (C) Cytotoxicity of NP and NP–tau on H4-swe cells after 48 h treatment by Trypan blue dye exclusion assay. Vehicle is phosphate buffer, and results are shown as the mean ± s.d. of two independent experiments. One-way ANOVA with Dunnett’s correction revealed no significant toxicity of NPs and NP–tau.

In conclusion, the aim of this work was to develop NPs with a surface chemistry mimicking the properties of the repeat domain of tau, which represents the region mostly contributing to the protein’s pathological transformation. We reasoned that by this camouflage the particles would acquire the ability to associate with the characteristic molecular assemblies of tau, that is, the aggregates and condensed states. We prepared fluorescent PEGylated SNPs functionalized with tau^4RD^ and observed that under conditions of in vitro aggregation for tau^4RD^ the presence of NP–tau had little influence on the kinetics of fibril formation and on the morphology of the filamentous aggregates, suggesting a weak interaction with aggregation intermediates. On the contrary, the nanoconjugates were found to be stably associated with the insoluble protein filaments. Moreover, NP–tau was recruited and concentrated into both polyU- and heparin-induced tau^4RD^ liquid condensates, exploiting multivalent electrostatic interactions with the polyanionic cofactor. Fluorescent labeling of NP–tau allowed us to visualize the phase-separated droplets by fluorescence microscopy and to follow their maturation, as demonstrated by the aggregation-linked dissolution observed for droplets obtained with heparin. We also showed that NP–tau could be internalized into human neuroglioma cells and did not exhibit cytotoxicity. Thanks to these fundamental properties, we propose that tau-functionalized, fluorescent NPs could find application as nanotracers to target and visualize tau assemblies or condensates, possibly contributing to illuminate crucial events during abnormal protein aggregation.

## Supporting Information Available

Experimental methods. Figure S1: Domain organization of tau. Figure S2: Heparin-induced droplets, control images (PDF)
