# Supplementary material for: Camouflaged Fluorescent Silica Nanoparticles Target Aggregates and Condensates of the Amyloidogenic Protein Tau
Source: Bioconjug Chem. 2022 Jun 10;33(7):1261–8. doi: 10.1021/acs.bioconjchem.2c00168 (PMC9305972; doi:10.1021/acs.bioconjchem.2c00168)
Supplement: Supplementary file 1 — bc2c00168_si_001.pdf [file bc2c00168_si_001.pdf]

SUPPORTING INFORMATION

Camouflaged fluorescent silica nanoparticles target aggregates and condensates of the amyloidogenic protein tau

Carlo Giorgio Barracchia,[†] Francesca Parolini,[†] Angela Volpe,[‡] Daniele Gori,[‡] Francesca Munari,[†] Stefano Capaldi,[†] Mariapina D'Onofrio[†] and Michael Assfalg[†]

[†] Department of Biotechnology, University of Verona, 37134 Verona, Italy

[‡] ACZON srl, Monte San Pietro, BO 40050, Italy

MATERIALS AND METHODS

Reagents

All reagents were purchased from Sigma Aldrich (St Louis, MO, USA) unless otherwise indicated.

Protein expression and purification

For the present investigation, recombinant protein tau^{4RD} (residues 244-372) and its variants tau^{4RD}(C322A) and tau^{4RD}(C291A/C322A) (also referred to as tau$^{4RD\Delta C}$), were expressed and purified as described previously.[1] Briefly, the proteins were expressed in BL21(DE3) cells grown in LB medium at 37 °C for 5 h, with 0.5 mM IPTG. Protein purification was performed by thermal treatment of the soluble bacterial extract (80-100 °C) and SP-ion exchange chromatography. Purified proteins were dialyzed against the working buffer (20 mM sodium phosphate, pH 7.4).

Synthesis of nanoparticles

AcZon nanoprobes are core-shell dye-doped silica nanoparticles synthesized through a micelle-assisted method.[2] The synthesis started with the formation of the micelles in water/1.5% v/v n-ButOH by stirring a solution of Brij58, Silane-PEG and the Rhodamine derivative for 1 h at room temperature. Next, the precursor MTMS (methyltrimethoxysilane) and Silane-PEG-NH_2 were added while stirring at 40 °C. After the addition of a 2.8% v/v solution of NH_3, the reaction was allowed to proceed under stirring at 40 °C for 48 h. NPs were first purified with Bio-beads (Biorad) to remove Brij58 and unreacted molecules, then dialyzed for 24 h. Amine groups on the surface were allowed to react with the crosslinker Succinimidyl trans-4-(maleimidylmethyl) cyclohexane-1-carboxylate (SMCC) (Setareh Biotech) by incubating 7 mL of NPs in 50 mM NaPi/1 mM EDTA pH=8 with 4.7 mg of SMCC (10 mM) for 1 h at room temperature, under stirring. The mixture was then purified by Desalting to eliminate unreacted SMCC using 50 mM MES/2 mM EDTA pH=6, thus obtaining NPs functionalized with maleimide groups. The NPs solution was concentrated using a centrifugation filter (50 kDa MWCO), quantified, and stored at 4 °C. The sample was characterized by spectrophotometry and spectrofluorometry. NP stock solutions were dialyzed against the working buffer with the addition of 1 mM EDTA.

Preparation of NP-tau conjugate

NP-functionalization was achieved incubating monomeric tau^{4RD}(C322A) in a NP-containing solution for 120 min at room temperature (stoichiometric ratio P/NP 10:1). Next, the solution was incubated for 60 min with 2 mM of reducing agent (DTT) in order to block the formation of dimers of the unreacted protein. The reaction product was then purified by dialysis (membrane MWCO = 100 KDa) to eliminate the unreacted protein. The final solution was concentrated using a centrifugation filter (3 kDa MWCO),

quantified, and stored at 4 °C. All these steps were analyzed by 15% SDS-PAGE under non-reducing conditions for 1 h at 220 V. The gel was scanned under UV-light and stained with Coomassie Blue.
The concentration of NPs was estimated by the absorbance at 566 nm using the extinction coefficient 55000 M^{-1} cm^{-1}. The concentration of protein conjugated to the NP was determined by the following formula:

$$C_{protein} = \frac{A_{280} - (0.21 x A_{566})}{\varepsilon_{protein}} \times \; dilution\,factor$$

UV-vis absorption spectra were recorded on a NanoDrop™ 2000 Spectrophotometer (Thermo Fisher), in 1 cm path-length quartz cuvette.

Fluorescence Spectroscopy

Fluorescence measurements were performed on a Jasco FP-8500 spectrofluorometer (Jasco, Easton, MD, USA) with a 1 cm path-length quartz cuvette, at room temperature. The excitation wavelength was 565 nm (slit width 5 nm), and emission spectra were collected in the range of 500-700 nm. Three spectra accumulations were averaged for each sample, and the spectrum of the buffer was considered as a blank and subtracted.

Dynamic Light Scattering (DLS) and Zeta (ζ) Potential Measurement

To investigate size distribution and ζ-potential of NP and NP-P, 20 µM of sample solution were prepared in working buffer. The measurements were performed using a Zetasizer Nano ZS (Malvern Instruments, Malvern, UK) at room temperature, operating at λ = 633 nm and equipped with a back scattering detector of 173°. Samples were allowed to equilibrate in the instrument holder for at least 10 min before starting measurements. Hydrodynamic diameter measurements were performed in triplicate. For ζ-potential measurements, seven replicate electrophoretic light scattering experiments were performed. The ζ-potential was derived from the electrophoretic mobility by means of the Henry equation.

NMR spectroscopy

NMR experiments were acquired on a Bruker Avance Neo spectrometer, operating at ^{1}H Larmor frequency of 600.13 MHz, equipped with a Prodigy TCI cryoprobe. NMR data were processed with Topspin 4.1.1 (Bruker). The samples were prepared in 20 mM potassium phosphate aqueous buffer at pH 7.4, also containing 7% D_2O.
One-dimensional ^{1}H-NMR experiments were acquired at 10 °C, with a standard pulse sequence incorporating the excitation sculpting water suppression scheme. A total of 32 transients were acquired over a spectral width of 16.0221 ppm, using a recycle delay of 1.2 s.

Agarose gel electrophoresis

Agarose electrophoresis was applied to investigate the electrophoretic mobility of samples. 1% agarose gel was prepared with and immersed in sodium phosphate buffer at pH 6. The samples were diluted by using running buffer that contained 10% glycerol, then loaded in the gel, and run for ~60 min at 120 V and 40 mA, using a HE 33 Mini-Sub Cell electrophoresis system (Amersham Biosciences). The gel was scanned under UV-light and stained with Coomassie Blue.

Dot blotting

About 3 μg of each sample were spotted on a PVDF membrane (Millipore). After blocking in TBS-T (0.1% Tween-20, 150 mM NaCl, 10 mM Tris at pH 8.0) with 5% (w/v) fat-free dry milk at room temperature, the membrane was washed three times with TBS-T and incubated with the anti-Tau, 359-373 primary antibody (BioLegend) specific for the tau^{4RD} domain (diluted 1:2000 in blocking buffer) at 4 °C overnight. Next, the membrane was washed three times with TBS-T and incubated with the anti-rat HRP-conjugated secondary antibody (BioLegend) diluted 1:2000 in blocking buffer for 2 h at room temperature. After washing three times with TBS-T, the blot was developed with ECL reagents (Pierce) on a ChemiDoc imager (BioRad).

Thioflavin-T Aggregation assay

A solution of tau^{4RD} was filtered through a 100 kDa MWCO filter (Sartorius Stedim Biotech GmbH, Göttingen, Germany) before starting the aggregation assay to remove pre-existing large oligomers and fibrils. The aggregation was induced by incubating the soluble protein (10 μM) with heparin (10 μM) in the absence (control) or presence of NP-tau (5 μM conjugated protein) in 20 mM sodium phosphate, 50 mM NaCl pH 7.4, 2 mM DTT. The kinetics of aggregation was monitored by measuring the fluorescence of the thioflavin-T (ThT, 10 μM) added to each sample in a 96-well dark plate (100 μL final volume for each well). Fluorescence measurements were performed using a microplate reader (TECAN Infinite M200 Pro, Tecan Group AG, Männedorf, Switzerland) at 30 °C for 48 h with cycles of 30 s of shaking (250 rpm, orbital) and 10 min of rest throughout the incubation. The fluorescence intensity was measured every 11 min (excitation, 450 nm; emission, 480 nm; bottom read). Error bars of fluorescence data correspond to standard deviations of at least four independent experiments.

Sedimentation assay

A sedimentation assay was performed on samples of tau^{4RD} and NPs. Samples containing 150 μM protein and/or 9 μM nanoparticles were prepared in 20 mM sodium phosphate pH 7.4, 50 mM NaCl, 2 mM DTT, then incubated with 37.5 μM heparin for 24 h at 37 °C without agitation. NP-tau were added either at t = 0 or after 24 h from sample preparation. In the latter case, the aggregated protein and nanoparticles were incubated for an hour. A control experiment was performed using $tau^{4RD\Delta C}$ and unconjugated NPs,

avoiding reaction of the protein with the maleimide groups on NPs. The solutions were then centrifuged at 15000 rpm for 40 min at 10 °C and washed, repeating the procedure four times (2× with buffer and 2× with water). The samples were finally examined under daylight or UV light (565 nm). The pellets were resuspended in water and analyzed by fluorimetry and TEM.

Far-UV Circular Dichroism (CD) Spectroscopy

CD measurements were carried out on a Jasco J-1500 spectropolarimeter equipped with a Peltier type thermostated cell holder (Jasco, Easton, MD, USA). Far-UV spectra (190–260 nm) were recorded at 25 °C at a scan rate of 50 nm min^{-1}, a bandwidth of 1 nm, and an integration time of 2 s, in 0.1 cm cuvettes. CD spectra were recorded on samples of tau^{4RD}, in the absence or presence of NP-tau (2:1 unbound:bound protein). Five spectra accumulations were averaged for each sample, and the spectrum of the buffer was considered as a blank and subtracted. The protein concentration was 6 µM.

Transmission Electron Microscopy (TEM)

For TEM measurements, samples were prepared as described for the ThT assay at the final volume of 100 µL and incubated for 48 h at 30 °C in static condition. Subsequently, one drop of solution (about 25 µL, 5 µM) in mQ H_2O was placed onto 400 mesh holey film grid; after staining with 2% uranyl acetate (for 2 min), the sample was observed with a Tecnai G^2 (FEI) transmission electron microscope operating at 100 kV. Images were captured with a Veleta (Olympus Soft Imaging System, Münster, Germany) digital camera using FEI TIA acquisition software (Version 4.0).

Visualization and analysis of condensates

Liquid-liquid phase separation of the investigated tau proteins was induced using either polyuridylic acid, poly(U) RNA (Sigma-Aldrich, P9528), or heparin (Sigma-Aldrich, H3393-100KU). 25 µM $tau^{4RD\Delta C}$ was mixed with a) 62.5 µg/ml poly(U) RNA in 25 mM Hepes pH 7.4 or b) 6.25 µM heparin, 30 mM NaCl in 20 mM NaPi pH 6.0. The use of the cysteine-free variant $tau^{4RD\Delta C}$ ensured better stability of the sample over time.
Fluorescent protein labeling was obtained with ALEXA Fluor 488 NHS ester (Thermo Fisher Scientific). Briefly, 6 mg $tau^{4RD\Delta C}$ (12 mg/ml) were incubated with 30 µl fluorescent reagent dissolved at 10 mg/ml in DMSO. The labeling reaction was carried out at room temperature for 1 h in shaking condition, and excess dye was removed by overnight dialysis (membrane MWCO = 6-8 kDa) in 20 mM sodium phosphate pH 7.4. Labelled proteins were then snap frozen and stored in aliquots at -20 °C, protected from light. For fluorescence imaging experiments, 1% Alexa488-labeled molecules were mixed with unlabeled protein.
At the indicated time of incubation, 7 µl of solution were spotted onto a microscope slide (Thermo Scientific, 26x76mm) and immediately covered with a circular coverslip. Images were acquired at Leica TCS SP5 AOBS microscope or on a Leica DM2500 optical microscope. Image analysis was performed to measure droplet size using FIJI Image J software (v2.0).

Cell culture

H4 APP-swe expressing cells (H4-swe cells) were grown in a humidified atmosphere of 5% CO_2 and passed in complete Dulbecco's modified Eagle's medium (DMEM) supplemented with 10% FBS (fetal bovine serum) (Aurogene), antibiotics (100 U of penicillin/ml, and 100 U of streptomycin/ml) and 2mM L-glutamine (Aurogene). Once 70–80% confluence was reached, cells were collected using trypsin, counted, and seeded for the experiments.

Cytofluorimetric analyses

H4-swe cells were treated in complete medium with different concentrations of NPs, replacing 10% FBS with 2% B27 Supplement (Thermo Scientific). After 48 h treatment, the cells were washed twice with PBS and then trypsinized, resuspended in phosphate buffered saline (PBS), and immediately analyzed with a flow cytometer. Dead cells and debris were excluded on the basis of forward (FSC) and side (SSC) scatter measurements, which confirmed a cell viability of >85% in each sample. Cellular uptake of NPs was verified exploiting the 561 nm laser (PE channel). Data were collected with the Becton Dickinson FACSAria Fusion and analyzed with FlowJo™ Software.

Confocal fluorescence microscopy of cells

For fluorescence confocal microscopy analyses, 50000 human H4-swe cells were seeded on glass coverslips coated with Poly-L-Lysine (Sigma-Aldrich, catalog #P8920) in a 24-multiwell microplate. After 48 h treatment with 2 µM NPs and NP-tau in complete medium (replacing 10% FBS with 2% B27 Supplement), cells were washed with PBS and fixed with ice-cold 4% PFA in PBS for 15 min at room temperature. After blocking with 5% bovine serum albumin and 5% Fetal Goat Serum and 0.2% Triton X-100 in PBS for 1 h at room temperature, cells were incubated with 2 µg/ml of anti β-tubulin III primary antibody (Merck) diluted in the blocking buffer for 2 h at room temperature. After 3 PBS washes, cells were incubated with 0.5 µg/ml anti-rabbit-Alexa Fluor 488 secondary antibody (Thermo Scientific) diluted in the blocking buffer for 1 h at room temperature. After PBS washes, nuclei were labelled with 1 µg/ml Hoechst diluted in PBS. Images were acquired using a confocal laser-scanning fluorescence microscope Leica TCS SP5 with a 63x HCX PL APO objective and analyzed with LAS AF software (Leica).

Cytotoxicity assay

Cell viability upon treatment with three different concentrations of NP or NP-tau and phosphate buffer (vehicle) was estimated using trypan blue exclusion assay. We note that the MTT assay is not suitable here due to NP absorbance near 570 nm (Figure 1B). Briefly, H4-swe cells were treated for 48 h in complete medium replacing 10% FBS with 2% B27 Supplement, as previously reported. After incubation, the medium was removed, cells were washed three times with PBS, trypsinized, and resuspended in 400 µl of

complete medium. The cell number was counted using the Countess™ and data were plotted and analyzed with GraphPad Prism 8.2 (GraphPad Software Inc., La Jolla, CA, USA). Data are expressed as a percentage of viability when compared with untreated cells, considered as 100% of viability. To ascertain statistically significant differences, One-way ANOVA with Dunnett's correction test was used. A conventional 5% level of statistical significance was considered.

REFERENCES

(1) Munari, F.; Barracchia, C. G.; Franchin, C.; Parolini, F.; Capaldi, S.; Romeo, A.; Bubacco, L.; Assfalg, M.; Arrigoni, G.; D'Onofrio, M. Semisynthetic and Enzyme-Mediated Conjugate Preparations Illuminate the Ubiquitination-Dependent Aggregation of Tau Protein. *Angew. Chem. Int. Ed.* **2020**, *59* (16), 6607–6611. https://doi.org/10.1002/anie.201916756.

(2) Pellegrino, C.; Volpe, A.; Juris, R.; Menna, M.; Calabrese, V.; Sola, F.; Barattini, C.; Ventola, A. Multiple Dye Doped Core-Shell Silica Nanoparticles: Outstanding Stability and Signal Intensity Exploiting FRET Phenomenon for Biomedical Applications. *J. Nanomater. Mol. Nanotechnol.* **2018**, *s6*. https://doi.org/10.4172/2324-8777.S6-003.

SUPPLEMENTARY FIGURES

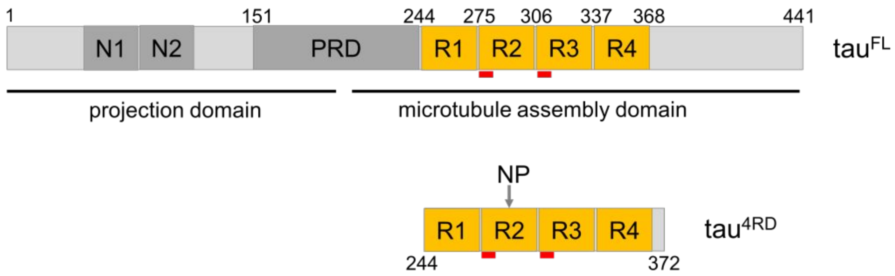

Figure S1. *Domain organization of tau^{FL} and tau^{4RD}*. R1-R4 are pseudo-repeats. Red bars indicate the position of the haxapeptide motifs PHF6*(^{275}VQIINK^{280}) and PHF6(^{306}VQIVYK^{311}). The conjugation position (Cys291) of tau^{4RD} with NP is indicated.

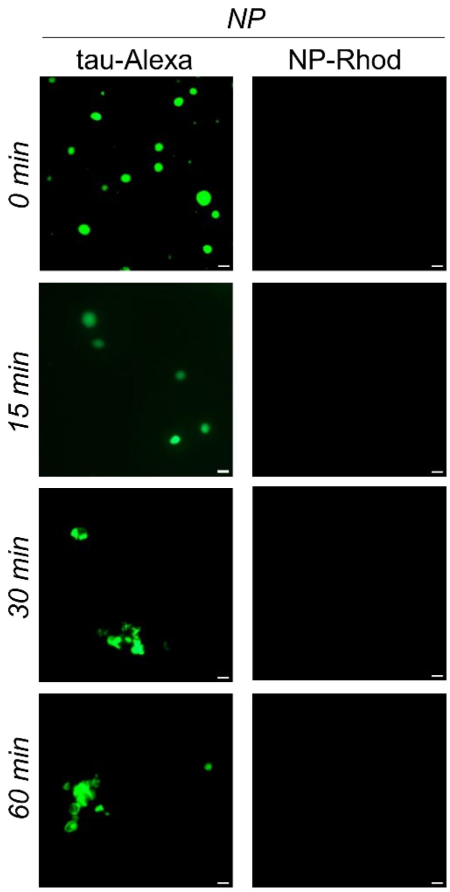

Figure S2. *Heparin-induced droplets*. Representative fluorescence microscopy images of condensates of tau$^{4RD\Delta C}$/heparin prepared in the presence of unconjugated NPs; images were acquired immediately after addition of protein and after 15 min, 30 min and 60 min of incubation; scale bars are 5 µm. Samples contained 25 µM protein and 6.25 µM heparin in 20 mM sodium buffer, 30 mM NaCl, pH 6; NPs were 0.3 µM.
